# Prevalence of *Cryptosporidium* spp. infection in rodents and chickens in Franceville, Gabon

**DOI:** 10.14202/vetworld.2024.1523-1529

**Published:** 2024-07-13

**Authors:** Patrice Makouloutou-Nzassi, Bernie Bouchedi, J. B. Mangombi-Pambou, Neil Michel Longo-Pendy, Nadine N’dilimabaka, Félicien Bangueboussa, Schedy Koumba, Anicet Mouity Matoumba, Larson Boundenga, Gael Darren Maganga, Rodrigue Mintsa-Nguema

**Affiliations:** 1 Unité de Recherche en Ecologie de la Santé, Centre Interdisciplinaire de Recherches Médicales de Franceville, Franceville, Gabon; 2 Département de Biologie et Ecologie Animale, Institut de Recherche en Ecologie Tropicale (IRET/CENAREST), Libreville, Gabon; 3 Department of Environment and Surveillance of Emerging and re-emerging diseases, Ecole Doctorale Régionale d’Infectiologie de Franceville, Franceville, Gabon; 4 Unité Emergence des Maladies Virales, Centre Interdisciplinaire de Recherches Médicales de Franceville, Franceville, Gabon; 5 Département de Biologie, Université des Sciences et Techniques de Masuku, Franceville, Gabon; 6 Department of Anthropology, Durham University, Durham, England, UK; 7 Department of Zootechnology, Institut National Supérieur d’ Agronomie et de Biotechnologies, Université des Sciences et Techniques de Masuku, Franceville, Gabon; 8 Department of Health and Environment, Université Libreville Nord, Libreville, Gabon

**Keywords:** *Cryptosporidium* spp, domestic chickens, Gabon, Oocysts, rodents

## Abstract

**Background and Aim:**

*Cryptosporidium* spp. members of the phylum *Apicomplexa* are obligate protozoan parasites capable of infecting various vertebrate hosts, including rodents and chickens. Infection caused by these parasites may lead to zoonotic diseases in humans. The aim of this study was to estimate the prevalence of *Cryptosporidium* spp. in rodents and domestic chickens sampled in Franceville, Gabon.

**Materials and Methods:**

Two hundred and eighty-five samples were collected, of which 185 samples were from rodents and 100 from domestic chickens. Microscopy after modified Ziehl-Neelsen staining and nested polymerase chain reaction targeting the small subunit (SSU) rRNA gene were used to examine *Cryptosporidium* spp.

**Results:**

The overall prevalence of *Cryptosporidium* oocysts was 55.8%, with a prevalence of 72.4% in rodents and 25.0% in domestic chickens. Molecular analysis showed that *Cryptosporidium* spp. were present in 4.0% of the samples. No significant correlation was observed between *Cryptosporidium* spp. carriage and sex or location in this study. These results indicate that *Cryptosporidium* spp. persist and circulate in the studied animal species in Franceville, Gabon.

**Conclusion:**

Infection with *Cryptosporidium* is very common in rodents and chickens in Franceville. The potential risk of human contamination cannot be ruled out. More research should be conducted to characterize *Cryptosporidium* species circulating in rodents and chickens in Gabon. Such studies are essential to better understand the epidemiology of this protozoan and its potential impact on public health.

## Introduction

*Cryptosporidium* species are obligate protozoan parasites belonging to the phylum *Apicomplexa* [1]. *Cryptosporidium* was first described by Ernest Edward Tyzzer in his laboratory in 1907 in mice and subsequently identified in various domestic and wild animals such as pets, livestock, and rodents [1–3]. The genus *Cryptosporidium* is globally distributed and includes several species isolated from many vertebrates, including humans, making a wide range of *Cryptosporidium* species potentially pathogenic to humans. *Cryptosporidium hominis* and *Cryptosporidium parvum* are the most implicated species in waterborne diarrhea outbreaks [2, 4]. In addition, approximately 25 species and 43 genotypes have been identified in bamboo rats, mice, voles, and many other wild rats [5]. Three species of *Cryptosporidium* have been found in poultry, with *Cryptosporidium meleagridis* being the third most common species causing human diarrhea [6, 7]. Isolation of *C. parvum* oocysts from the digestive tract of rats without intestinal inflammation or diarrhea in the feces suggests that rodents serve as reservoirs for *Cryptosporidium* spp. [8].

*Cryptosporidium* is an intestinal protozoan parasite recognized as a human pathogen since 1976 [9]. It causes a zoonotic disease called cryptosporidiosis, which occurs when *Cryptosporidium* invades the brush border of enterocytes. The World Health Organization considers cryptosporidiosis a neglected disease [10]. However, cryptosporidiosis can be life-threatening for immunocompromised individuals, children under 2 years of age, and older adults [11]. The main symptom of cryptosporidiosis is diarrhea, which is usually self-limiting in immunocompetent humans but can be life-threatening in vulnerable populations. Over 90% of 165 waterborne outbreaks have been linked to *Cryptosporidium* contamination [12]. Notable outbreaks have included Milwaukee (Wisconsin, USA, 1993) [13], Sète (France, 1998), Dracy-le-Fort (France, 2001), and Divonne-les-Bains (France, 2003) [14]. The most recent outbreak involving small mammals occurred in the United Kingdom in 2008, where a genotype from a species isolated from rabbits was identified [15].

To the best of our knowledge, no study has been conducted on cryptosporidiosis in rodents and domestic chickens in Gabon. There have been a few research studies on cryptosporidiosis in humans, pets such as dogs or cats, and wildlife in Gabon. Oyegue-Liabagui *et al*. [16] and Manouana *et al*. [17] have identified *C. hominis* and *C. parvum* in the diarrheal stools of children under 5 years of age in hospitals in Franceville (at 19%) and Lambaréné (at 12.9%), respectively. These studies show the circulation of these two species in Gabon. However, there have been limited studies on the circulation of *Cryptosporidium* in potential reservoirs. To date, the only studies conducted in Lambaréné have shown a relative risk of cryptosporidiosis in human contact with an infected pet (dog or cat) [18]. In addition, studies on great apes in the Lopé and Moukalaba-Doulou national parks have revealed the presence of *C. hominis* and *C. parvum* in gorillas [19]. All these studies conducted in Gabon have shown that *Cryptosporidium* circulates in the wild, especially in great apes, as well as in urban environments. Other studies have shown that rodents and chickens can carry species and genotypes of *Cryptosporidium* that have definite zoonotic potential [6-8]. However, little information is available on the occurrence of cryptosporidiosis in rodents and chickens in Gabon, as well as the contribution of these animals to the maintenance of the epidemiological foci.

Therefore, the aim of this study was to estimate the prevalence of natural *Cryptosporidium* infection in rodents and domestic chickens in Gabon to assess and mitigate the risk of possible cryptosporidiosis outbreaks.

## Materials and Methods

### Ethical approval

Rodents were captured with the agreement of the mayor of the city of Franceville and with the consent of the owner of the concession where the capture occurred. All sampling procedures were approved by the “Comité National d’Ethique pour la Recherche” Ethics Committee under the number: Prot n 0020/2013/SG/CNE. Similarly, sampling of chickens was done with the permission of the owners in the different districts of the city of Franceville.

### Study period and location

This study was conducted from November 2021 to April 2022 at the Laboratory of Unité de Recherche en Ecologie de la Santé, Centre Interdisciplinaire de Recherches Médicales de Franceville.

### Sample from animal collections (rodents and chicken)

This study is a component of the protocol established by Mangombi *et al*. [20] for the screening of rodent pathogens in the city of Franceville. Fecal samples were collected from rodents captured in wire traps (Tomahawk and Sherman traps) placed in various districts of the city, including peripheral districts (Mbaya, Sable, Yene, Djakana, Mingara, and Bapili) and central districts (Centre Interdisciplinaire de Recherches Médicales de Franceville [CIRMF], Potos, Ombele, and Ongouenié) following Mangombi et *al*. [20]. All rodent trapping procedures and organ sampling techniques were performed in accordance with the methods described by Mangombi *et al*. [21]. In addition, fecal samples were collected from domestic chickens in the same districts. Poultry owners were randomly selected after presenting the study objectives and the significance of understanding the pathogens that their poultry might carry. Only owners who provided their consent were included in this study. The hens and roosters were captured at dusk when they were baited in the kitchen or lazaretto with rice. Rodent fecal samples were placed in Eppendorf tubes (Eppendorf AG, 22331 Hamburg, Germany) and stored in a freezer at –80°C until laboratory analysis. Fecal collection from domestic chickens involves gently inserting a swab into the cloaca. The droppings were collected and placed in a 15 mL Falcon tube containing saline. For each sample (rodent or chicken), we recorded information regarding the capture location or housing conditions (in a pen or not, inside kitchens, etc.), species, sex, and identification.

### Sample preparation and modified Ziehl-Neelsen (MZN) staining

Formalin-ethyl acetate sedimentation for stool concentration: One gram of feces was homogenized with 4 mL of distilled water and filtered through a mesh funnel. The filtrate was collected in a 15-mL conical tube, and saline solution (0.9%) was added to fill the tube. The tube was centrifuged at 3000× *g* for 10 min. The supernatant was discarded, and 9 mL of 10% formalin was added to the sediment. The mixture was vigorously mixed. Finally, 4 mL of ethyl acetate was added, and the tube was capped and shaken in an inverted position. After shaking, the tube cap was slowly removed to gradually release the pressure, followed by another round of centrifugation at 3000× *g* for 10 min. Four layers were observed: ethyl acetate, debris plug, formalin, and the sediment pellet. The three aqueous phases are gradually discarded. Smears were prepared from 100 μL pellets, air-dried, and fixed with methanol. Staining was performed using the following steps: (a) staining with 1% carbol-fuchsin for 60 min, (b) thorough rinsing in tap water, (c) decolorization in acid alcohol (2% H_2_SO_4_ in ethanol) for 20 s, (d) thorough rinsing in tap water, (e) counterstaining with 5% malachite green for 1 min, and (f) thorough rinsing and air drying. Microscopic examination was conducted to detect the presence of oocysts by systematically scanning the slides using a brightfield microscope (Danaher, USA) with at least a 40× lens. The presence of oocysts was confirmed using a high-power objective lens (e.g., 100×). *Cryptosporidium* spp. Oocysts appear pink to red with spherical to ovoid bodies against a blue background [22].

### DNA extraction and molecular detection

*Cryptosporidium* DNA was extracted from the fecal sample using the ROBOKLON stool DNA kit (EURx Ltd. 80–297 Gdansk Poland ul. Przyrodnikow 3, NIP 957-07-05-191 KRS 0000202039, www.eurx.com.pl), following the manufacturer’s procedure. *Cryptosporidium* was detected by nested polymerase chain reaction (PCR) at the small subunit (SSU) rRNA gene [23, 24]. Amplicons were visualized in 1.5% agarose gels with GelRed (Biotium Inc., Fremont, CA, USA) following electrophoresis.

### Statistical analysis

Microsoft Excel 2016 (Microsoft Corp., Washington, USA)) and R software R.4.3.2 (R Foundation for Statistical Computing, Vienna, Austria) [25] were used for the analysis. Categorical variables are presented as frequencies, and prevalence is accompanied by p-values. Fisher’s exact test was used to compare the proportions, and the significance level was set at 5%.

## Results

### Study population

Two hundred and eighty-five samples, consisting of 185 rodents and 100 domestic chickens, were selected. Most of the study population (approximately two-thirds) was female, with 107 female rodents and 70 female chickens. This resulted in a gender ratio of 0.54. *Rattus rattus* was the most abundant species, accounting for 166 individuals or 82.18% of the rodent population. One of the invasive species was *R. rattus*, while the other four were native species. Only one chicken species, *Gallus gallus domesticus*, was included in this study. Table-1 summarizes the characteristics of the overall study population.

**Table-1:** Overall data on studied animal species and sampling location.

District	Rodent							Chicken	
	
	M	F	Cr	Lem	Mus	Pra	Rat	M	F
Peripheral									
Bakou								2	12
Bapili								7	5
Djakana								2	6
Mbaya	15	25		1	2		37		
Makana								1	1
Mingara								7	9
Mangoungou	13	12			2		23	1	11
Ngoungoulou								4	9
Yene	14	21		3	6	1	25	1	4
Central									
Ongougnie									
Sable	9	19					28		
CIRMF	2	4					6		
Ombele	5	12		1			16		
Potos	20	14	1		2		31	1	4

M=Male, F=Female, Cr=*Crycetomys* spp., Lem=*Lemniscomys*, Mus=*Mus nannomys* spp., Pra=*Praomys*, *Rat=Rattus rattus*

### Occurrence of *Cryptosporidium*

Table-2 summarizes the results for positive samples from Franceville, Gabon, including the animal types and sampling origin. *Cryptosporidium* DNA was detected in 11 fecal samples, indicating an infection rate of 4.0%. *Cryptosporidium* infection prevalence was slightly higher in males (4.6%) compared to females (3.4%) (p > 0.05). A higher proportion of samples collected from peripheral districts (4.2%) tested positive for *Cryptosporidium* than samples collected from central districts (2.6%) (p > 0.05). *Cryptosporidium* infection was found in 10 out of 185 rodents and in 1 out of 100 *G. gallus domesticus* species. Using MZN staining, 55.8% (159/285) of the samples were positive for *Cryptosporidium* oocysts, 72.4% (134/185) of rodents, and 25% (25/100) of chickens. Overall, regardless of the animal species, a higher percentage of females (57.6%) was infected than males (52.8%), although this difference was not statistically significant (p > 0.05). In addition, the prevalence of infection was higher in central districts (65.3% vs. 52.4%, p > 0.05) than in peripheral districts.

**Table-2:** Infestation rates of *Cryptosporidium* spp. according to animal species and location.

Location	No. of samples examined	Diagnosis of *Cryptosporidium*					

		Microscopy		p-value	PCR amplification		p-value
	
		%(n)	%(n)		%(n)	%(n)	
Rodent							
Peripheral	128	71.1 (91)	72.4 (134)	p=1	6.25 (8)	5.4 (10)	p=1
Central	57	75.4 (43)			3.5 (2)		
Chicken							
Peripheral	82	23.2 (19)	25 (25)		1.2 (1)	1.0 (1)	
Central	18	33.3 (6)			0.0 (0)		

### Infestation rates of *Cryptosporidium* spp. according to rodent species

In general, five rodent species were found to be carriers of *Cryptosporidium* oocysts. *Cricetomys* spp. (1/1, 100%), *Praomys* spp. (1/1, 100%), *R. rattus* (125/166, 75.3%), *Lemniscomys* spp. (3/5, 60%), and *Mus nannomys* spp. (4/12, 33.33%) tested positive for *Cryptosporidium* oocysts. Following PCR DNA amplification, it was determined that only *Lemniscomys* spp. (1/5, 20%) and *R. rattus* (9/166, 5.42%) were carriers of *Cryptosporidium* spp. parasites.

### Geographical distribution of *Cryptosporidium* spp. infection according to the sites studied in the city of Franceville

**Figure-1:**
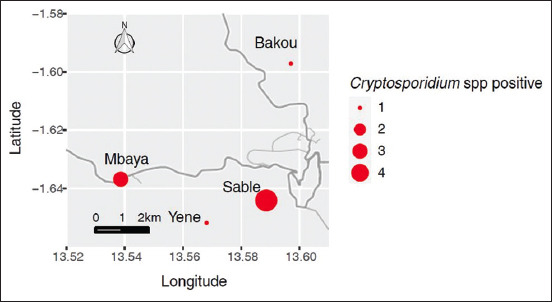
Geographical distribution of Cryptosporidium spp. in investigated animals according to sample collection sites in Franceville [Source: The map was generated using ggmap packages from R version 4.3.3 with maps.stamen.com].

For this purpose, the mapping shows the number of cases based on the districts from which the samples were obtained. Peripheral districts of the city have the highest number of recorded infections. The majority of infected animals are found in these peripheral areas. Furthermore, the prevalence of *Cryptosporidium* spp. infection varied across the peripheral districts of Franceville city, with Sable (4), Mbaya (3), Yene (1), and Bakou (1) being the most affected (Figure-1).

## Discussion

This cross-sectional study is the first of its kind conducted in Gabon and, overall, it is the first in the Central African sub-region to involve both wild and domestic animals sharing the same environment. Other studies conducted in Gabon by Langhout [19], Oyegue-Liabagui *et al*. [16], and Manouana *et al*. [17] have focused on *Cryptosporidium* spp. in great apes in the Lopé and Moukalaba-Doudou national parks and humans in the cities of Franceville and Lambaréné but do not provide insight into the potential reservoirs in several animal species, especially those close to humans. In this study, microscopic approaches were used to detect *Cryptosporidium* oocysts. This observation corroborates well with the literature on the use of MZN stain to detect coccidial oocysts in rodents and poultry [26, 27]. Using this technique, we found prevalence rates of 72.4% and 25% in rodents and domestic chickens, respectively. This high prevalence suggests that rodents play a significant role in endoparasite carriage. Indeed, rodents are already recognized as carriers or reservoirs of most zoonotic agents involved in human and animal outbreaks [5], which is worrisome for the potential occurrence of *Cryptosporidium* gastroenteritis in Gabonese settings, especially since the rodents in our study were captured in peri-domiciliary districts. In addition, since local chickens are domestic poultry with which people live almost permanently, such an infestation rate (25%) increases the risk borne by households where these animals are housed close to their homes, sometimes without enclosures. The oocysts are emitted by fecal clearance, making the droppings a definite source of contamination for other species, including humans.

The overall prevalence of *Cryptosporidium* after MZN in rodents in this study was much higher than 54% reported by Salem [28] or 1.5% reported by Ayinmode *et al*. [29]. Likewise, it is not comparable to the 19.8% worldwide prevalence of *Cryptosporidium* spp. in rodents [5]. The high prevalence reported in this study could be attributed to the geographical area, ecology, and sample size. Our study revealed a higher prevalence of *Cryptosporidium* in rodents than in chickens (p > 0.05). This trend may be related to different exposure levels among the animal species studied in this study. Rodents are small- to medium-sized mammals with short reproductive cycles, large litters, and morphological and biological adaptations to different lifestyles (e.g., terrestrial, subterranean, and gliding) and environments (e.g., semiaquatic, aquatic, or dry biotopes). All of this leads to increased contact with infectious pathogens such as *Cryptosporidium*, which is found in all environments [30].

*Cryptosporidium* infection in chickens from various African countries has been reported in previous studies. The prevalence varies according to the country: 4.5% in Tunisia [31], 7.4% in Nigeria [32], 9%–69% in Algeria [33], 10.5% in Malawi [34], and 34% in Algeria [7]. The infection rate reported in the present study is comparable to the trend of previous studies. This slight difference can be attributed to the sample size or the method used to analyze samples. However, unlike some of these studies, this species has not been identified in this study. The challenge in identifying *Cryptosporidium* species can be attributed to the fact that nested PCR in the SSU ribosomal RNA gene can detect all *Cryptosporidium* species [23, 35]. In contrast to microscopy, the additional advantage of utilizing PCR lies in its ability to differentiate *Cryptosporidium* spp. This capability is typically achieved through a combination of restriction fragment length polymorphism analysis, DNA sequencing, or other molecular techniques [36]; however, these methods were not used in the present study.

In this study, *Cryptosporidium* infection was found to be more prevalent in animals residing in peripheral districts than in the central districts. This discrepancy can be attributed to various factors, such as sample size, ecological factors, water contaminated with feces, proximity of habitation, and higher population density. These factors contribute to the transmission of the parasite between animals and humans [37]. The most frequently reported risk factors for *Cryptosporidium* infection include overcrowding, diarrhea, poor drinking water quality, contact with animals, open defecation or insufficient sanitation facilities, and breastfeeding. Animal contact and open defecation primarily account for most *Cryptosporidium* cases in low- and middle-income countries [38]. The peripheral areas of Franceville, where the density of the animals studied appears to be higher, are of particular concern. These areas have the potential to become hotspots for the dissemination of *Cryptosporidium* infection. From this point of view, *Cryptosporidium* transmission routes are particularly significant due to the close interactions between residents, rodents, and local chickens. There is an opportunity for direct transmission if these animals are present indoors or in breeding practices near residential areas. In addition, rodents, which serve as potential reservoirs of *Cryptosporidium*, may contribute to the contamination of the local environment, including water sources and surfaces that are frequently touched. Another potential factor for the spread of infection is the practice of feeding chickens by residents, which is common in this region.

Our research supports previous studies by Li *et al*. [39] and Vanathy *et al*. [40], showing a higher prevalence and risk of *Cryptosporidium* in female *equus* than in male *equus*. Our results suggest that females are more susceptible to *Cryptosporidium* than males. This may be due to a weak immune system, especially after giving birth, and their tendency to live in burrows during pregnancy. For example, female rodents are less active during gestation and remain in their holes [41], making them more vulnerable to *Cryptosporidium* infection from contact with the ground [37]. In the current study, the positive rates of *Cryptosporidium* obtained using molecular techniques were comparatively higher than those obtained by other investigators, such as 1.5% found in Nigeria [29], 0.7% found in Iran [42], but lower than the rate of 11.5% found in China [27] or 14.8% observed in Brazil [43]. Similarly, the rate of *Cryptosporidium* positivity after PCR amplification (4.0%) was comparatively lower than that after MZN (55.8%) in this study. This contradicts previous studies by Elsafi *et al*. [44], Morgan *et al*. [45], and Van den Bossche *et al*. [46], who used microscopy to report prevalence instead of molecular tools, which usually reported lower *Cryptosporidium* prevalence. According to Da Cunha *et al*. [43], differences in prevalence could be related to differences in sample collection (pool vs. individual sample), sample origin (tissue vs. feces), age of animals, density of animals, or housing conditions. However, molecular techniques are generally more sensitive than microscopy for the detection of infection. Nevertheless, sometimes, microscopy shows a high detection rate, as in this study. Possible explanations include the presence of PCR inhibitors in feces (bilirubin, bile salts, and complex polysaccharides), the failure of oocyst disruption and DNA extraction, or insufficient oocyst concentration in the samples subjected to extraction [22].

In this study, among all the rodent species screened, *R. rattus* were more infected by *Cryptosporidium* (75.3% after MZN and 5.42% after PCR amplification), which is within the reported range of 0.7%–100% for *Cryptosporidium* prevalence in rodents [5]. Previous studies have also reported a high prevalence of *Cryptosporidium* in this rodent species: 56% in the United Kingdom [47], 30.5% in Iran [48], and 8.2% in Australia [49]. Moreover, these findings highlight that the prevalence of *Cryptosporidium* in rodents varies greatly.

## Conclusion

This epidemiological study estimated the prevalence of *Cryptosporidium* spp. in peridomiciliary rodents and domestic chickens in Gabon. *Cryptosporidium* spp. is present in these two animal populations studied in Franceville, Gabon. Consequently, the risk of contamination for humans and other animals through the environment or direct contact should not be underestimated in the Gabonese context. Nonetheless, this study has limitations. Although nested PCR method is widely recognized for its sensitivity and specificity, the variance in prevalence between microscopy and molecular analysis suggests a more precise and adjusted protocol. Therefore, we could not identify the specific *Cryptosporidium* species that circulated within our screened population. Further research involving the characterization of parasites from various environmental locations, livestock, drinking, and recreational water sources is necessary to gain a better understanding of the direct or indirect transmission routes between rodents, livestock, and humans and to elucidate the role of rodents and domestic chickens in the zoonotic transmission of *Cryptosporidium*.

## Authors’ Contributions

PMN: Conceived and designed the study. BB, JBMP, NMLP, FB, SK, and AMM: Carried out the study. PMN, BB, and NMLP: Analyzed the data. PMN, BB, JBMP, NMLP, and NN: Wrote the manuscript. LB, GDM, and RMN: Supervised the study. All authors have read, reviewed, and approved the final manuscript.
